# Therapeutic potential of FGF19 in combatting osteosarcopenia: effects on muscle strength and bone health in aged male mice

**DOI:** 10.1093/jbmrpl/ziaf157

**Published:** 2025-10-01

**Authors:** Hao Zhang, Priyanka Garg, Bérengère Benoit, Claudie Pinteur, Stéphanie Chanon, Aurélie Vieille-Marchiset, Emmanuelle Loizon, Alice Beau, Norbert Laroche, Jérôme Ruzzin, Hubert Vidal, Laurence Vico, Maura Strigini

**Affiliations:** Université Jean Monnet Saint-Étienne, Mines Saint-Étienne, INSERM, SAINBIOSE U1059, F-42023 Saint-Étienne, France; Université Jean Monnet Saint-Étienne, Mines Saint-Étienne, INSERM, SAINBIOSE U1059, F-42023 Saint-Étienne, France; Laboratoire CarMeN, INSERM U1060, INRAe U.1397, Université Claude Bernard Lyon1, Fr-69310 Pierre Bénite, France; Laboratoire CarMeN, INSERM U1060, INRAe U.1397, Université Claude Bernard Lyon1, Fr-69310 Pierre Bénite, France; Laboratoire CarMeN, INSERM U1060, INRAe U.1397, Université Claude Bernard Lyon1, Fr-69310 Pierre Bénite, France; Laboratoire CarMeN, INSERM U1060, INRAe U.1397, Université Claude Bernard Lyon1, Fr-69310 Pierre Bénite, France; Laboratoire CarMeN, INSERM U1060, INRAe U.1397, Université Claude Bernard Lyon1, Fr-69310 Pierre Bénite, France; Laboratoire CarMeN, INSERM U1060, INRAe U.1397, Université Claude Bernard Lyon1, Fr-69310 Pierre Bénite, France; Université Jean Monnet Saint-Étienne, Mines Saint-Étienne, INSERM, SAINBIOSE U1059, F-42023 Saint-Étienne, France; Department of Molecular Medicine, Faculty of Medicine, Institute of Basic Medical Sciences, University of Oslo, Oslo, Norway; Laboratoire CarMeN, INSERM U1060, INRAe U.1397, Université Claude Bernard Lyon1, Fr-69310 Pierre Bénite, France; Université Jean Monnet Saint-Étienne, Mines Saint-Étienne, INSERM, SAINBIOSE U1059, F-42023 Saint-Étienne, France; Université Jean Monnet Saint-Étienne, Mines Saint-Étienne, INSERM, SAINBIOSE U1059, F-42023 Saint-Étienne, France

**Keywords:** FGF19, osteosarcopenia, bone microarchitecture, muscle strength, aging

## Abstract

Osteosarcopenia, characterized by the coexistence of osteopenia/osteoporosis and sarcopenia, represents a significant health concern in geriatrics, with an increased risk of falls and fractures. The enterokine fibroblast growth factor 19 (FGF19) was recently shown to prevent muscle weakness in preclinical models. This study investigated the therapeutic potential of FGF19 in mitigating bone and muscle deterioration in aged male mice. Twenty-one-month-old C57BL/6 male mice received daily injections of human recombinant FGF19 (0.1 mg/kg) for 21 days. Histological and functional analyses revealed a shift toward larger muscle fibers in FGF19-treated mice as well as an increased muscle strength, without affecting muscle mass. In parallel, X-ray microtomography showed that FGF19 had no overt negative impact on bone, with a range of modest, site-specific, and opposing effects. In the distal femur metaphysis FGF19, it reduced cortical thickness, but significantly increased bone cross-sectional area, with an overall increased polar moment of inertia, a geometrical parameter linked to favorable mechanical properties. It also elevated cortical bone porosity in the same region. There were no significant effects on trabecular bone or cortical bone parameters in the proximal femur side at the lesser trochanter level nor at the femoral midshaft or in the tibia. In the L2 vertebra, cortical porosity decreased. Histomorphometry of trabecular bone and analysis of transcriptional output of selected genes in femurs revealed only minor changes in bone cellular activities and gene expression after three weeks of treatment. In conclusion, FGF19 treatment increased muscle strength in aged male mice, without negatively impacting aging bone.

## Graphical Abstract

**Graphical Abstract:**
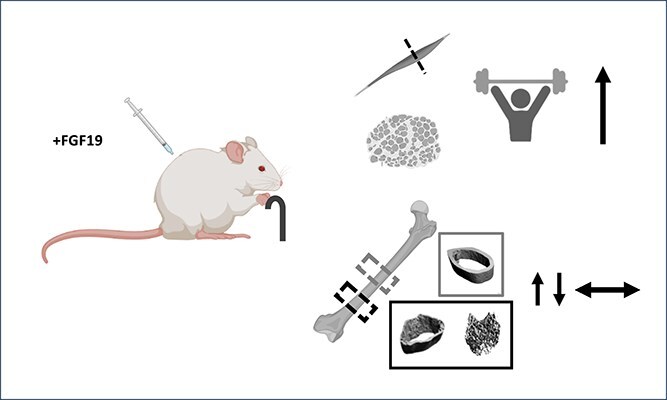

## Introduction

Aging is a common risk factor for many chronic musculoskeletal pathologies, including osteoporosis and sarcopenia. Osteosarcopenia combines these two conditions and locks them in a vicious circle.^1^ Lower BMD and bone microarchitectural deterioration make the skeleton fragile and fracture-prone.^2^^,^^3^ Lower muscle mass, strength and function increase the risk of falls, which can lead to bone fractures.^4^ Limited physical activity due to frailty decreases the mass and quality of both muscles and bones.^5^ In the case of fracture, the prolonged mechanical unloading of the musculoskeletal system imposed on bedridden individuals can further worsen this scenario. Lastly, in osteosarcopenia, the molecular cross-talk between the two organ systems, based on the exchange of hormonal and paracrine signals, is altered: it becomes pro-catabolic rather than pro-anabolic or homeostatic, further weakening both organs.^6^ Osteosarcopenia severely diminishes the quality of life and it curbs the ability to live independently, especially in the elderly. It results in a significant burden on the individuals affected and in a high socio-economic cost to society at large.^7^

Currently, no agency-approved drug treatments for osteosarcopenia are available. It is hoped that solutions could be found by simultaneously targeting both sides of the osteosarcopenia vicious circle. Bone is a dynamic tissue: bone-forming osteoblasts and bone-resorbing osteoclasts continually remodel bone, through repeated alternate cycles. Among the approved antiresorptive agents (ie, drugs for osteoporotic patients that target osteoclast activity and that exert an anti-catabolic action in the bone) the RANKL inhibitor denosumab has also shown positive effects on hand-grip strength and falls in non-randomized clinical studies.^8^^,^^9^ These promising results are supported by data in preclinical models,^10^ but they need to be confirmed in randomized clinical trials specifically addressing osteosarcopenia. Although extremely rare, severe side effects of denosumab have been reported,^11^ therefore alternative treatments would be welcome.

Recently, our group and others showed that treatment with fibroblast growth factor 19 (FGF19) can counter the sarcopenic and cachectic phenotypes in preclinical models of muscle weakness in vivo*.*^12–16^ It can indeed protect skeletal muscle from atrophy caused by glucocorticoid treatment, obesity or aging,^12^^,^^15^^,^^17^ and is also beneficial when renal function is severely impaired.^14^ Interestingly, an epidemiological study in postmenopausal women with osteoporosis showed that these patients had significantly lower levels of FGF19 compared to healthy women.^18^

Members of the FGF family are extensively involved in regulating cell growth and development, tissue repair, and metabolic processes.^19^ FGF19 (FGF15 in rodents) is a non-typical FGF that belongs to the endocrine class of FGFs, lacking the heparin-binding domain.^20^ At the signal transduction level in muscle cells, FGF19 acts by binding to the FGF receptor4 (FGFR4)/β-klotho complex, which in turn activates the extracellular signal-regulated kinase 1/2 (ERK1/2) and the mammalian target of rapamycin (mTOR) pathways, thus initiating its hypertrophic effects.^12^

Cool et al. reported that FGFR4 is expressed in pre-osteoblasts and osteoblasts in neonatal mouse calvaria, suggesting that it might be involved in osteogenesis, although its role in bone homeostasis remains unclear.^21^ Furthermore, it was proposed that FGFR1, an alternative receptor for FGF19, may participate in osteogenesis since FGFR1 deficiency in immature osteoblasts increases proliferation and delays differentiation and matrix mineralization, while in differentiated osteoblasts, the lack of FGFR1 enhances mineralization.^22^ Guo et al. showed that FGF19 regulates osteoblastic differentiation in the MC3T3-E1 pre-osteoblastic cell line, potentially through the Wnt/β-catenin signaling pathway, and that it may control osteoclastogenesis indirectly, through the osteoblast-to-osteoclast OPG/RANKL axis.^23^ Li et al. detected beta-klotho expression in bone marrow mesenchymal stem cells (BMMSC) and bone monocyte-macrophage precursors (BMM), but not in MC3T3 and BMMSC-derived osteoblasts instead.^24^ Because of the above and of the strong cross-talk between muscles and bones,^6^^,^^25^ it is tempting to speculate that, beyond having an effect on muscle, FGF19 could be involved in bone metabolism, by directly or indirectly acting on bone cells.

To date, very few studies have simultaneously addressed the impact of an FGF19-based treatment on muscle and bone in osteopenia models. Recently, we showed that in a chronic kidney disease (CKD) model FGF19 significantly protects against muscle wasting and attenuates hepatic inflammation, but it fails to effectively protect against bone loss.^14^ Pereira et al. found that FGF19 increases the tibial weight, but has no effect on the tibial length in Wistar rats with cerebral palsy, while also having beneficial effects on muscles.^13^ Concentrating only on the skeletal side, Guo et al. investigated the protective effects of FGF19 on bone loss induced by obesity in a high-fat diet-fed obese mouse model and in mouse pre-osteoblasts (MC3T3-E1) treated with palmitic acid.^23^ They concluded that FGF19 alleviates obesity-induced bone loss in mice. These studies suggest that the action of FGF19 on bone may depend on specific pathological conditions or other regulatory factors and may operate through multiple mechanisms.

We hypothesized that FGF19 could help counteract aging-associated bone loss and muscle atrophy. To test this hypothesis, we administered human recombinant FGF19 daily over a period of 21 days to 21-month-old C57BL/6 male mice and assessed the protective effects of the treatment on bone and skeletal muscle parameters.

## Materials and methods

### Experimental design and animals

Thirty-four 21-month-old C57BL6 male mice (Envigo) were randomly assigned to two groups. One group (*n* = 17) received daily subcutaneous injections of human recombinant FGF19 at 0.1 mg/kg (R&D System) in vehicle every morning for 21 days, while the other group (*n* = 17) was injected with vehicle (phosphate buffer solution, 0.1% of bovine serum albumin). We selected 0.1 mg/kg and 21 days of injections because our previous studies found that these conditions significantly prevented muscle loss, including on mice of similar age.^12^^,^^14^

Each mouse was singly housed and maintained under standard conditions on a 12/12 h light/dark cycle and with access to water and regular chow diet ad libitum (Rod16-R, LASvendi). Once a week, mice were weighed and individual food intakes were calculated. At the end of the experimental procedure, mice were euthanized with ketamine/xylazine. Liver and skeletal muscles (gastrocnemius and tibialis anterior (TA)) were weighed, and all collected muscles including soleus were snap-frozen in liquid nitrogen and stored at −80 °C. Soleus and TA muscles from the contralateral leg were embedded in OCT-tissue freezing medium (General Data) for histological analysis. Right femurs, right tibias, and lumbar vertebrae were harvested, fixed for 36 h in 4% formaldehyde solution at 4 °C, then conserved in 80% ethanol and employed for micro-CT (μCT) analysis and histomorphometry. Left femurs were stored in liquid nitrogen for real-time PCR analysis. All experimental protocols were approved by the local ethics committee (“comité d’éthique en expérimentation animale de la Région Rhône-Alpes, Lyon, France”, CECAPP #LS_2019_005) and in accordance with the guidelines laid down by the French Ministry of Agriculture (n°2013-118) and the European Union Directive for the protection of animals used for scientific purposes of September 22, 2010 (2010/63UE). All methods are reported in accordance with the ARRIVE guidelines. All efforts were made to minimize the number of animals used and their suffering.

### Muscle strength testing

After 13 days of treatment, forelimb grip strength was assessed using a standard grip strength device (Grip Strength Test Meter, Bioseb). Mice were grasped by the proximal tail and placed on the grip device and then gently pulled until the grip was lost. Between 3 and 5 trials were performed for the grip strength measure for each mouse. The average of all recorded values was considered as the maximum strength. The normalized strength was the maximum value divided by the mouse body weight (therefore expressed in g/g).

### Histological analysis of muscles

Fixed samples of TA and soleus muscles were cryosectioned (10-μm-thick sections taken at the mid-belly of the muscles) and processed for immunostaining, as described previously.^12^ Briefly, sections were blocked for 1 hour at room temperature and incubated overnight at 4 °C with a rabbit anti-laminin antibody (L9393, Sigma), followed by incubation with a fluorescent secondary antibody (AlexFluor Goat anti-Rabbit IgG Alexa Fluor 594-A11012, ThermoFisher). The 10x magnification images were taken using a BX63 Olympus upright microscope. The Fiji software was configured to take into account only the transverse fibers with a Feret ratio strictly up to 0.5. The muscle fiber area was measured in μm^2^.

### Micro-computed tomography analysis of bones ex vivo

For the microtomographic (μCT) analysis, the formalin-fixed and ethanol-dehydrated right femur, right tibia, and second lumbar (L2) vertebra were scanned using the VIVA CT40 tomograph (Scanco Medical), in accordance with standard guidelines.^26–28^ Data acquisition settings included a voltage of 70 keV, a current of 114 μA, and an isotropic voxel size of 10.5 μm. Three-dimensional reconstructions of the bones were generated: a smoothing sigma (σ) of 1.2, support value of 2, and a threshold of 195 for trabecular bone and a smoothing sigma (σ) of 0.8, support value of 1, and a threshold of 260 for cortical bone.

The structural parameters of trabecular bone in the femur, tibia, and lumbar vertebra included bone volume/total volume (BV/TV, %), trabecular thickness (Tb.Th, mm), trabecular number (Tb.N, 1/mm), and trabecular separation (Tb.Sp, mm). They were measured in a set of 150 sections excluding the primary spongiosa, above (proximal to) the distal growth plate for the femur, a set of 150 sections below (distal to) the proximal growth plate for the tibia, and from all sections between the two growth plates of the L2 vertebra, respectively.

Cortical bone parameters include cortical thickness (Ct.Th, mm), total cross-sectional area inside the periosteal surface (Tt.Ar, mm^2^), cortical area (Ct.Ar, mm^2^), marrow area (Ma.Ar, mm^2^), cortical porosity (Ct.Po, %), and tissue mineral density (TMD, mg HA/mm^3^). The structural parameters of cortical bone were obtained through contouring the inner and outer boundaries of each cortical section of a set of 60 sections and were determined by integrating values from each transverse section. ROI for cortical bone was automatically contoured using an edge detection software tool. For the tibia, only the mid-diaphysis, corresponding to a position at 50% of the total length of the tibia, was examined. For the femur, three specific regions were analyzed: the distal metaphysis, the mid-diaphysis, and the proximal side at the small trochanter level, representing 75%, 50%, and 25% of the total length of the femur, respectively. The polar moment of inertia (pMOI, mm^4^) in the distal metaphysis was calculated from the maximal (Imax) and minimal (Imin) areal moments of inertia: pMOI = Ixx + Iyy, mm^4^.

The results for the structural parameter cross-sectional area of the femur cortical bone at the distal metaphysis (total cross-sectional area inside the periosteal surface) were further validated through a detailed two-dimensional slice-by-slice analysis. This analysis was performed at the VIVA CT40 tomograph: the region of interest was larger than what is described above, yet similarly identified, that is, starting above the femur distal growth plate and avoiding the primary spongiosa, then extending 450 slices proximally (ie, toward the diaphysis). Bone cross-sectional area of each slice was directly extracted as part of the comprehensive 3D analysis for this region offered by the analysis suite provided by the tomograph manufacturer. Slices were taken at each voxel on the z-axis (10.5 μm apart). Values were averaged for the treatment (FGF19) and control (vehicle) groups and displayed in a heatmap graph obtained with GraphPad Prism software.

### Bone histomorphometry

Histomorphometric analyses were performed on right femurs after μCT analysis according to a method developed in this laboratory.^29^ Briefly, the samples were fixed, dehydrated in absolute acetone, and embedded mineralized in methylmetacrylate-based medium at low temperature. Seven-micrometer-thick sections were obtained using a tungsten-carbide-bladed microtome (Leica SM2500E) then stained with a Modified Goldner staining, or for tartrate-resistant acid phosphatase (TRAP) activity with toluidine blue counterstaining.

Bone volume/total volume (BV/TV, %), trabecular thickness (Tb.Th, μm), trabecular separation (Tb.Sp, μm), and trabecular number (Tb.N, 1/mm) measurements were done on Modified Goldner stained sections using the Bone v.3.50 automatic image analyzer software (Explora Nova) and a Leitz DMRB microscope (Leica). All analyses were performed in a 2.5 mm × 2.5 mm area of the distal femur metaphysis, on 6 non-consecutive sections. TRAP and Toluidine Blue stained sections were analyzed via a software developed in-house, coupled to a Summasketch III digitizing table (Summagraphics) and an Axio Scope.A1 microscope (Carl Zeiss). Osteoclast number (Oc.N) and osteoclast surface/bone surface (Oc.S/BS, %) were calculated using TRAP-stained sections. Osteoblast surface/bone surface (Ob.S/BS, %) was calculated using toluidine blue stained sections.^27^^,^^29^

### RNA isolation, reverse transcription, and quantitative real-time qPCR in muscle samples

Total RNAs from soleus muscles were extracted using TRI Reagent (Sigma Aldrich). Purity and concentration of RNA were determined using NanodropOne (Ozyme) and quality checked using Bioanalyser (Agilent). First-strand cDNAs were synthesized from 1 μg of total RNAs using PrimeScript RT kit reagent kit (Perfect Real Time) (Takara Bio Europe). Real-time qPCR assays were performed on 1/20 or 1/60 diluted cDNA using TB Green Premix Ex Taq (Tli RNaseH plus) (Takara Bio Europe) with Rotor-Gene 6000 (Qiagen), as previously described.^14^^,^^30^ TATA-box binding protein (*Tbp*) was used as a reference gene to normalize the results. Results are the ratio of target mRNA levels to *Tbp* mRNA levels and are expressed in arbitrary units. Primer sequences are listed in Table S1.

### RNA isolation, reverse transcription, and quantitative RT-qPCR in femur samples

After euthanasia, whole left femurs were carefully dissected and immediately stored in cryotubes at −80 °C. The bone marrow was not removed from the femurs. The whole femur was crushed to powder before RNA extraction, using tungsten balls in a liquid-nitrogen cooled Mikro-Dismembrator S mixer mill (Sartorius). Total RNA was extracted by TRI Reagent (Sigma) and then purified with a Qiagen pure tissue kit (RNeasy Plus Mini Kit, Qiagen). The RNA quality was assessed by Tapestation 4150 automated electrophoresis platform (Agilent Technologies), followed by Ribogreen assay (Quant-iT RiboGreen RNA Assay Kit, Invitrogen, Life Technologies) for accurate RNA quantification. Reverse transcription of total RNA to synthesize complementary DNA (cDNA) was carried out with 1 μg of RNA using the iScript cDNA Synthesis Kit (Bio-Rad). Quantitative real-time (qRT) PCR was conducted using the CFX96 Real Time System (Bio-Rad) with the Light Cycler FastStart DNA Master plus SYBR green I kit (Roche Diagnostics). The starting quantities (SQ) of the targeted genes were calculated based on a standard range via the CFX Manager software (Bio-Rad). All SQ data were normalized to the expression of β-actin. Primers sequences are listed in Table S1.

### Statistical analysis

Statistical analysis and graphical representations were performed using GraphPad Prism 9. Mann–Whitney U Tests were used to compare the two groups of mice. Values of *p* < .05 were considered statistically significant. Data are presented as the mean ± SEM.

## Results

### Impact of FGF19 on body parameters and muscle strength in aged male mice

**Figure 1:**
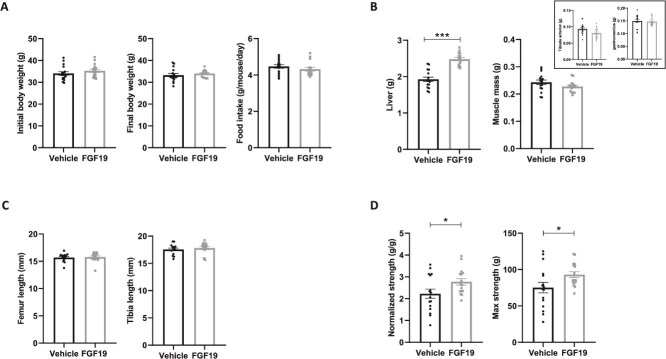
FGF19 treatment enhances muscle strength and liver weight. (A) Panel of figures showing, from left to right, the initial body weight of the mice (in g), the body weight after 21 days of treatment with FGF19 or vehicle solution (in g), and the food intake measured throughout the treatment period (in g/mouse/d). (B) Tissue weights collected at sacrifice, with liver weight shown on the left (in g) and combined gastrocnemius and tibialis anterior (TA) muscle weight on the right (in g). Inset shows tibialis and gastrocnemius weights, separately. (C) Length of the femurs and tibias collected at sacrifice (in mm). (D) Parameters representing muscle strength during the grip strength test, with normalized muscle strength (relative to body weight, in g/g) on the left, and maximum strength on the right (in g). Data are presented as mean ± SEM. All data were analyzed using Mann–Whitney U tests. ^*^ indicates *p* < .05, and ^***^ indicates *p* < .001. Mice treated with the vehicle solution are represented in black, while mice treated with FGF19 (0.1 mg/kg) are represented in gray.

As illustrated in Figure 1, treatment with FGF19 over a period of 3 weeks did not induce any changes in body weight or food intake (Figure 1A). We observed a significant increase in liver weight in the treated mice and a maintenance of muscle mass (weights of the gastrocnemius muscle and TA muscle combined, Figure 1B). Tibia length and femur length were not different between the two groups (Figure 1C). Grip strength tests conducted after 13 days of treatment revealed a significant increase in muscle strength (+25%) in treated mice compared to controls (Figure 1D).

### FGF19 treatment shifts skeletal muscle fiber distribution toward larger fibers without affecting expression of atrophy markers

**Figure 2:**
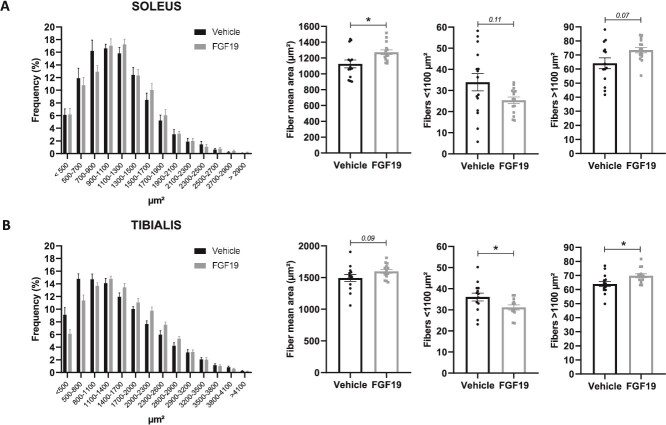
FGF19 treatment shifts muscle fiber distribution toward larger fibers. Panel of figures representing muscle fiber analysis in the soleus (A) and tibialis anterior (TA) (B) muscles of mice. From left to right: distribution frequency of muscle fibers by cross-sectional area (in μm^2^), mean fiber area (in μm^2^), percentage of fibers with an area smaller than 1100 μm^2^, and percentage of fibers with an area greater than 1100 μm^2^. This value corresponds to the peak of the unimodal distribution for control groups for both muscles, shown in the histograms. Therefore, it represents the most common fiber size under basal conditions in aged mice. Data are presented as mean ± SEM. All data were analyzed using Mann–Whitney U tests. ^*^ indicates *p* < .05. Mice treated with the vehicle solution are represented in black, while mice treated with FGF19 (0.1 mg/kg) are represented in gray.

Histological analyses were conducted on the soleus and TA muscles to measure the impact of FGF19 on the distribution of muscle fibers according to their size (Figure 2A and B). As previously observed,^12^ the distribution curves for both muscles showed a distinct rightward shift along the x-axis for the mice treated with FGF19. This indicated that the FGF19-treated mice had muscles containing fewer small fibers in favor of larger fibers (the number of fibers did not vary with treatment—data not shown). These observations were confirmed by the fact that the average fiber areas in the soleus and TA muscles were increased in the FGF19 mice (*p* < .05 in soleus, *p* = .09 in TA). Additionally, smaller fibers (with a surface area < 1100 μm^2^) were less numerous in the muscles of FGF19 mice compared to vehicle mice (*p* < .05 in TA, *p* = .11 in soleus), and the opposite trend was observed for larger fibers (*p* < .05 in TA, *p* = .07 in soleus).

Sarcopenia is classically associated with increased expression of gene markers for atrophy and inflammation. In our study, we investigated the potential effects of FGF19 treatment on the expression of four atrophy markers (Figure S1) and two muscle inflammation markers (Figure S1) in the soleus muscles of mice. The results showed large intragroup variability in the mRNA levels of these markers, which precluded demonstrating any impact of FGF19 on atrophy and inflammation pathways in this study.

### FGF19 treatment does not alter femur trabecular bone

**Figure 3:**
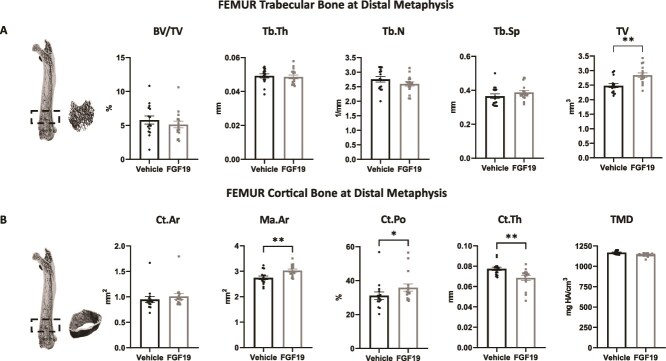
FGF19 treatment affects bone microarchitectural parameters at the distal metaphysis of the femur. Panel of figures representing bone microarchitecture analysis of the distal femur at the level of the metaphysis: trabecular bone (A) and cortical bone (B). Parameters that describe trabecular bone are not affected by FGF19 treatment. Changes in total volume and marrow area suggest alterations in bone size at this site, accompanied by changes in cortical porosity and thickness. Abbreviations: BV/TV, bone volume/total (bone volume fraction); Tb.Th, trabecular thickness; Tb.N, trabecular number; Tb.Sp, trabecular separation; TV, total volume of trabecular bone; Ct.Ar, cortical area; Ma.Ar, marrow area; Ct.Po, cortical porosity; Ct.Th, cortical thickness; TMD, tissue mineral density. Data are presented as mean ± SEM. All data were analyzed using Mann–Whitney U tests. ^*^ indicates *p* < .05; ^**^ indicates *p* < .01. Mice treated with the vehicle solution are represented in black, while mice treated with FGF19 (0.1 mg/kg) are represented in gray.

**Figure 4:**
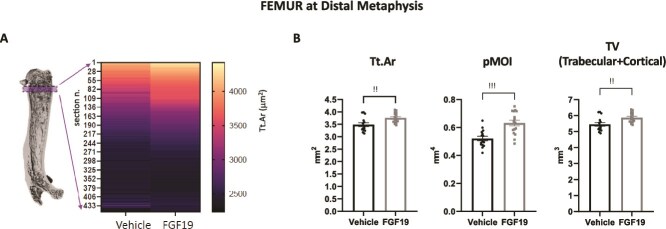
Enlarged distal femur in FGF19-treated mice. (A) Heat map showing bone total cross-sectional area inside the periosteal surface distal to the growth plate in vehicle injected vs FGF19 injected aged mice. Bone cross-sectional area is represented from larger cross-sectional area (light) to narrower cross-section (dark) in μm^2^. FGF19 injected aged mice have larger bone cross-sectional area for up to roughly 200 slices proximal to the growth plate, when compared to vehicle injected aged mice as evident from the heat map. This analysis was done using 2D slice by slice analysis of distal metaphysis below the growth plate, with each slice taken at 10.5 μm interval. Distal to the top. (B) Additional bone microarchitectural parameters at the distal metaphysis of femur: Tt.Ar (total cross-sectional area inside the periosteal surface); pMOI (polar moment of inertia); total volume (defined by the outer periostal surface). In (B) data are presented as mean ± SEM. All data were analyzed using Mann–Whitney U tests. ^**^ indicates *p* < .01; ^***^ indicates *p* < .001. Mice treated with the vehicle solution are represented in black, while mice treated with FGF19 (0.1 mg/kg) are represented in gray.

Bone microarchitectural parameters were assessed by ex vivo μCT. First, we considered the effects on femur trabecular bone. FGF19 treatment did not significantly affect the trabecular bone structural parameters at the distal metaphysis of the femur. There were no significant differences between the FGF19-treated group and the vehicle group in terms of bone volume/total volume (BV/TV), trabecular thickness (Tb.Th), trabecular number (Tb.N), and trabecular separation (Tb.Sp) (Figure 3A). Although classic descriptors of trabecular bone microarchitecture were not altered by FGF19 treatment, including BV/TV, it was noticed that the volume occupied by the trabecular bone was larger, with increases of both BV (not shown) and TV (Figure 3A), which prompted us to further analyze the distal femur (Figure 3B and 4).

### FGF19 treatment affects the size of femur distal metaphysis

In the analysis of cortical bone at distal metaphysis, the bone total cross-sectional area inside the periosteal surface (Tt.Ar) was significantly increased in the FGF19-treated group (*p* < .01, Figure 4B), indicating an increase in the overall size of the bone in this region. On the other hand, cortical bone porosity (Ct.Po) was significantly increased (*p* < .05) and Ct.Th was significantly decreased in the FGF19-treated group, with Ma.Ar also increased (Figure 3B). Importantly, there were no significant differences in cortical bone surface area (Ct.Ar) in this region of the bone, showing that the same amount of cortical bone tissue is present (Figure 3B). Finally, TMD of the cortical bone at the distal metaphysis was unchanged between control and FGF19-treated group (Figure 3B).

The finding of enlargement of bone cross-sectional area in the distal femur metaphysis was further supported by a detailed 2D slice-by-slice analysis, extended to an even larger portion of the distal femur for a total of 450 slices, 10.5 μm apart, starting from the distal growth plate and moving toward the diaphysis. Figure 4A shows a heatmap representation of bone cross-sectional areas of the distal femur in vehicle injected vs FGF19 injected mice: bone cross-sectional area in μm^2^ is color-coded from high values (light yellow) to lower values (dark purple). The analysis showed a progressively diminishing effect when moving from the most distal to less distal positions (ie, from the distal metaphysis to the diaphysis): FGF19 injected aged mice had larger bone cross-sectional area up to roughly 200 slices below the growth plate when compared to vehicle injected aged mice as shown in the heatmap.

The polar moment of inertia pMOI is used to estimate bone strength and torsional rigidity from μCT parameters. In the distal femur metaphysis, FGF19 treatment significantly increased pMOI (Figure 4B). This suggests that FGF19 may help to enhance the torsional strength and overall stability of the bone.

Taken together, these results suggest that FGF19 treatment significantly affected certain structural parameters at the distal metaphysis of the femur.

### FGF19 treatment does not alter femur proximal side and mid-diaphysis

**Figure 5:**
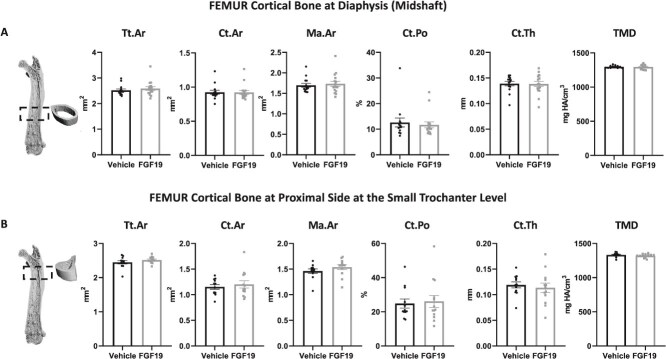
No effect of FGF19 treatment on bone microarchitectural cortical parameters at the midshaft and at the proximal trochanter in the femur. Panel of figures representing bone microarchitecture analysis of cortical bone at the femur diaphysis (midshaft, A) and at a proximal site at the level of the trochanter (B)**.** No significant difference could be recorded, indicating no effect on overall bone size, nor on cortical parameters at these two sites: Tt.Ar (total cross-sectional area inside the periosteal surface); Ct.Ar (cortical area); Ma.Ar (marrow area); Ct.Po (cortical porosity); Ct.Th (cortical thickness); TMD (tissue mineral density). Data are presented as mean ± SEM. All data were analyzed using Mann–Whitney U tests. Mice treated with the vehicle solution are represented in black, while mice treated with FGF19 (0.1 mg/kg) are represented in gray.

We additionally considered the effects on cortical bone at mid-diaphysis and at the level of the small trochanter in the proximal femur. FGF19 treatment did not significantly affect the cortical bone structural parameters at these two sites (Figure 5A and B). Specifically, there were no significant differences between the FGF19-treated group and the vehicle group in terms of cortical bone area (Ct.Ar), Ma.Ar, cortical bone porosity (Ct.Po), cortical bone thickness (Ct.Th), and bone TMD. The overall size of the bone was also unchanged, with the cross-sectional area (Tt.Ar) unchanged.

### FGF19 treatment does not impact tibia cortical and trabecular bone

The results of tibia cortical bone analysis at mid-diaphysis showed no significant differences between the FGF19-treated group and the vehicle group in terms of cortical bone area (Ct.Ar), cortical bone thickness (Ct.Th), cortical bone porosity (Ct.Po), Ma.Ar, and TMD (Figure S2A). Similarly, total cross-sectional area inside the periosteal surface (Tt.Ar) was unaffected, an indication that overall bone size was not altered by FGF19 treatment at this site.

The results of trabecular bone analysis at the proximal metaphysis of the tibia (Figure S2B) also showed no significant differences between the FGF19-treated group and the vehicle group in terms of bone volume/total volume fraction (BV/TV), trabecular thickness (Tb.Th), trabecular number (Tb.N), and trabecular separation (Tb.Sp). Contrary to what was observed in the distal femur, unaltered total volume (TV) and bone volume (BV) showed a lack of effect of FGF19 treatment on bone size in the proximal tibia.

These results suggest that FGF19 treatment did not impact tibia bone microarchitecture.

### FGF19 treatment lowers cortical porosity and has no impact on trabecular bone in the L2 vertebra

The analysis of trabecular and cortical bone parameters in the L2 vertebra are presented in Figure S3. For trabecular bone (Figure S3A), there were no significant differences between the FGF19 treatment group and the vehicle group in terms of bone volume fraction (BV/TV), trabecular thickness (Tb.Th), trabecular number (Tb.N), and trabecular separation (Tb.Sp).

The cortical bone porosity (Ct.Po) was slightly, yet significantly, lower in the FGF19-treated group compared to the vehicle group (*p* < .05) (Figure S3B). Because of the effect of FGF19 on the cross-sectional area at the distal femur metaphysis, we also evaluated the total cross-sectional area (Tt.Ar) at the mid-vertebral position, but no significant differences were detected (Figure S3B).

### Impact of FGF19 on bone cellular parameters

**Figure 6:**
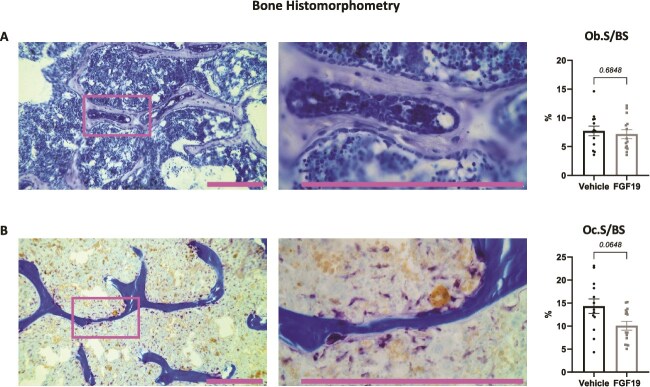
Bone histomorphometry on frontal sections of femurs shows no difference in bone forming and resorbing activities after three weeks of FGF19 treatment. (A) Goldner staining (left); quantification of osteoblast surface (Ob.S) over bone surface (BS) (right). (B) TRAP-staining (left and center); quantification of osteoclast surface (Oc.S) over BS (right). (A and B) The image at the center is a higher magnification (taken with 40X vs 10X objective) of the one on the left, corresponding to the area defined by the colored rectangle on the left. Scalebars correspond to 250 μm. Data in graphs are presented as mean ± SEM. All data were analyzed using Mann–Whitney U tests. Mice treated with the vehicle solution are represented in black, while mice treated with FGF19 (0.1 mg/kg) are represented in gray.

Histomorphometry analysis was carried out on frontal sections of distal femur to look into possible changes in bone resorption and formation activities upon FGF19 treatment. Figure 6A and B display the microscopy images of osteoblasts and osteoclasts along with their respective quantitative analyses. The results indicated that FGF19 did not affect osteoblast surface (Ob.S/BS). A decrease in active osteoclastic surface (Oc.S/BS) in the FGF19 treatment group was noted, although it did not reach statistical significance (*p* = .0648), even if osteoclast number over bone surface (Oc.N/BS, 1/μm) was actually higher in the FGF19 group (*p* = .001; vehicle group: median: 3.63, min-max: 3.19-4.49, mean: 3.72, standard error of the mean: 0.10; FGF19 group: median: 4.64, min-max 4.10-10.43, mean: 5.59, standard error of the mean: 0.58). Osteoblast numbers were not determined.

### Impact of FGF19 on the gene expression of bone metabolic markers

We further investigated the effects of FGF19 treatment on the expression of bone metabolic marker genes, from samples of whole femurs collected at sacrifice, after 3 week of treatment. In the analysis of genes related to osteoblast/osteocyte activity, the results showed no significant differences between the FGF19 treatment group and the vehicle group in the expression of osteocalcin (OCN), osteoprotegerin (OPG), osterix/SP7 (Osx), sclerostin (Sost), Runx2, and alkaline phosphatase (*Alp*) genes (Figure S4A). In the analysis of genes related to osteoclast activity, no significant differences appeared between the FGF19 treatment group and the vehicle group in the expression of *c-FOS*, ATP-ase H+ transporting V0 subunit D2 (*Atp6v0d2*), tartrate-resistant alkaline phosphatase (*TRAP*), receptor activator of nuclear factor kappa-B ligand (*Rankl*) (*alias TNFSF11*), dendrocyte expressed seven transmembrane protein (*Dcstamp*), matrix metalloproteinase 9 (*MMP9*), Cathepsin K (*Ctsk*) genes, nor for the *Rankl/OPG* ratio; there was an increase in *Rank* gene expression in the FGF19 treatment group (with *p* = .0562, Figure S4B). In the analysis of genes related to adipogenesis and osteogenesis regulation (Figure S4C), FGF19 treatment significantly increased the expression of peroxisome proliferator activated receptor gamma (*PPARγ*) gene (*p* < .0001), while there were no significant differences in the expression of CCAAT enhancer binding protein alpha (*C/EBPα*) and CCAAT enhancer binding protein beta (*C/EBPβ*).

## Discussion

Osteoporosis and sarcopenia are prevalent in older adults and are correlated with morbidity and mortality.^1^^,^^7^^,^^31^ Currently, no FDA-approved drugs are available for the treatment of sarcopenia and osteosarcopenia.^1^^,^^7^^,^^32^ FGF19 and its murine orthologue FGF15 are members of the endocrine FGF family and are secreted by ileal enterocytes in response to bile acids.^33^ Previous studies, including our own, have demonstrated the protective effect of FGF19 on the muscle tissue.^12–15^ Meanwhile, in recent years, more research has focused on the effect of FGF19 on bone health.^23^^,^^34^ FGFR4, receptor for FGF19, and FGFR1, an alternative receptor of FGF19, have been reported to participate in osteogenesis,^21^^,^^22^^,^^35^ by transducing signals from a variety of FGF superfamily members. The mRNA for beta-Klotho, the obligatory co-receptor for FGF19 and 21, was also detected in bone.^24^ One can thus envisage that FGF19 could have direct effects on bone. However, the simultaneous assessment of FGF19’s effects on muscle and bone in animal models of osteosarcopenia has rarely been reported.^14^

Based on the protective effect of FGF19 against muscle loss observed in our previous research, considering the strong crosstalk between muscle and bone, and knowing that relevant receptors are expressed in bone, we hypothesized that FGF19 could have direct and/or indirect beneficial effects on bone tissue and bone cells in the clinically relevant osteosarcopenic model of aging. To test this, we administered recombinant human FGF19 to evaluate its effects on the bones and muscles of male mice in very advanced age (21-month-old), where muscle and bone loss is important.

Our results showed that continuous FGF19 treatment for three weeks significantly increased muscle strength in aged male mice. This was not secondary to an increase in body size or in feeding. We confirmed that large fibers in the FGF19-treated group were more numerous, as in our previous work.^12^ These results align with other studies conducted both in vivo and in vitro*.*^12–15^^,^^36^^,^^37^ However, unlike in other reports, the protective effect of FGF19 appeared not to impact muscle mass in the current study. This discrepancy might be partially explained by the different pathophysiological basis of the sarcopenia of each model, and hence the ability of FGF19 to counter muscle mass deficits, and/or by variations in doses and treatment durations across different pathological conditions. In a CKD model, 18 days of FGF19 treatment at 0.1 mg/kg effectively increased skeletal muscle size in 6-week-old mice and reduced gene expression of hepatic inflammatory markers and myostatin.^14^ In contrast, in the current study, none of the examined markers of muscle atrophy or inflammation were significantly affected by FGF19 treatment in aged mice. In 13-week-old genetically obese (*ob/ob*) mice, a higher dose of FGF19 (1.0 mg/kg) administered over 7 days resulted in significant improvements in skeletal muscle strength, weight, and mean TA area, with fiber hypertrophy.^12^ Age-related muscle loss likely involves multifactorial mechanisms, such as mitochondrial dysfunction and neuromuscular junction degeneration,^38–40^ which may not be fully addressed by FGF19 treatment alone.

Skeleton-wise, FGF19 did not affect the femur cortical parameters at the level of the small trochanter and at the midshaft. At the femur distal metaphysis, cortical TMD, trabecular bone volume ratio (BV/TV) and microarchitectural parameters remained unchanged. This is consistent with our previous findings in a CKD model, where FGF19 showed no effect on skeleton.^14^ However, at the femur distal metaphysis, we observed an increase in TV and BV values suggesting a larger bone at this site in treated animals. Enlargement of this region was confirmed by an increased bone total cross-sectional area inside the periosteal surface (Tt.Ar). Micro-CT 2D slice-by-slice analysis corroborated and extended this observation, showing that FGF19-injected aged mice had larger femur bone cross-sectional area when compared to vehicle injected mice, for about 2 mm of the distal metaphysis in the femur starting from the growth plate (roughly 12% of total femur length). The Ct.Ar, defined as the bone surface between the outer periosteal perimeter and the inner endosteal perimeter, remained unchanged in the FGF19-treated group, while pMOI increased. Polar moment of inertia is a geometrical parameter from which biomechanical properties can be extrapolated, reflecting the resistance of a bone’s cross-section to torsional deformation, with greater values indicating enhanced ability to withstand stresses induced by torque. It is believed that, during aging, periosteal apposition and the ensuing increase in pMOI serve as partial compensation to the other alterations in bone microarchitecture and the ongoing degradation of bone quality afflicting the elderly.^41–43^ This appears to be more important in the FGF19-treated animals, signifying a regulation of material distribution, resulting in structural optimization. Our bone histomorphometry analysis on frontal sections of distal femur, that took a snapshot of bone cell activities after three weeks of treatment, did not give clear hints as to how these changes come about, since no statistically significant changes were identified for osteoblastic and osteoclastic activities.

Although the increased cross-sectional area (Tt.Ar) and pMOI are indicative of stronger bones, the increase in Ct.Po in this distal region would signify, in itself, a locally weaker bone. Nevertheless, even small increases in the cross-sectional area (Tt.Ar) confer improved resistance to compressive forces and exponentially increased resistance to bending forces and are greater contributor to bone strength than changes in Ct.Po.^41^^,^^44^^,^^45^ None of the other tested micro-architectural parameters in the mid- or proximal femur, proximal tibia or L2 vertebra were affected, except the L2 Ct.Po that is slightly decreased in treated mice.

The action of FGF19 on bone remains unclear, particularly in the context of osteoporosis or osteosarcopenia. In an epidemiological study on 150 postmenopausal women, Zhao et al. reported a positive correlation between FGF19 serum levels and BMD.^18^ In a distinct epidemiological study involving 374 postmenopausal women with osteoporosis, serum FGF19 showed instead no significant correlation with skeletal parameters.^46^ On a cellular level, Guo et al. suggested that in vitro FGF19 potentially regulates osteoblastic differentiation in MC3T3-E1, through the Wnt/β-catenin signaling pathway, and osteoclastogenesis indirectly, through the OPG/RANKL axis.^23^ FGF19 responding cells are expected to express beta-Klotho. Li et al. reported detectable expression of co-receptor beta-Klotho in BMMSC and BMM precursors, but not in BMMSC-derived osteoblasts nor MC3T3 cells.^24^ The two studies leave open the question of which cells can be direct targets of FGF19 in bone.

FGF19 and FGF21, two of the endocrine FGFs, signal through distinct FGF receptors, but share the co-receptor beta-Klotho.^20^^,^^47^ Both preclinical work and clinical observational findings suggest that the two hormones have distinct effects on bones: contrary to what we and others^23^ observe with FGF19 injections in mice, Wei et al. found that chronic FGF21 over-expression driven by a transgene as well as daily injection with 1 mg/kg FGF21 for 2 week can induce severe bone loss and alter RANKL/OPG ratio in mice.^48^ Li et al. showed that FGF21 negatively regulates bone homeostasis, possibly via FGFR1 and beta-Klotho-mediated direct signaling in BMMSC and BMM cells.^24^ It is speculated that in bone FGF21 promotes the differentiation of adipocytes at the expense of osteoblasts.^48^^,^^49^ Consistently, and differently from what observed for FGF19, the larger epidemiological study mentioned above found that elevated serum FGF21 levels were significantly negatively associated with bone strength parameters at the intertrochanteric region of the hip.^46^

In the present study, gene expression analysis by RT-qPCR demonstrated that FGF19 significantly upregulated the expression of *PPAR-γ* (peroxisome proliferator-activated receptor gamma), a key regulator of adipocyte differentiation, in aged bone. FGF19 may promote the differentiation and accumulation of adipocytes through increased PPAR-γ expression. If this occurred at the expense of osteoblast differentiation, according to the model of competitive osteoblast vs adipocyte differentiation from common precursors,^10^^,^^49^ it would be expected to bear negatively on bone health and micro-architectural parameters, something we did not observe.

The present study has some limitations. First, the absence of young control group (eg, 4-month-old mice) limits the comparative analysis of age-specific effects. Second, while the 3-week FGF19 treatment showed significant muscle protection effect, the duration of the study may not have been sufficient to capture all potential long-term effects on the skeleton in very old male mice. Third, the study only focused on structural parameters in specific bone regions without assessing the impact of FGF19 on bone mechanical properties. Future studies should incorporate mechanical testing^50^ to comprehensively evaluate its effects on bone health. In addition, to better characterize the effect of FGF19 on muscle, it would have been valuable to measure total lean mass using a body composition analyzer. Unfortunately, such equipment was not available at the time the study was conducted. Finally, we were not able to investigate the FGF19-induced intracellular signaling pathways in skeletal muscle, particularly the ERK/mTOR axis, due to limitations in the number of animals available for this study.

In conclusion, the novelty of this study is the simultaneous assessment of FGF19 treatment on both skeletal and muscular systems in an aged mouse model of osteosarcopenia. We provide evidence that FGF19 treatment increase muscle strength in 21-month-old male mice with no striking negative or positive impact on bone. The observed effects on bone, like on the geometry of the femur distal metaphysis, remain rather modest, while others have found a stronger impact in an HFD model.^23^ It is thus possible that the effects of FGF19 on bone may depend on specific pathological states or other regulatory factors. The role of FGF19 in regulating bone homeostasis remains to be further investigated using other models of bone loss*,* such as ovariectomy (OVX) or chronic disuse, such as hindlimb unloading.

## Supplementary Material

FGF19_Aging_Osteoarcopenia-Zhang_Garg_ziaf157

## Data Availability

The data supporting the findings of this study are available upon fair request by e-mail to the corresponding author.
