# Supplementary material for: Therapeutic potential of FGF19 in combatting osteosarcopenia: effects on muscle strength and bone health in aged male mice
Source: JBMR Plus. 2025 Oct 1;9(12):ziaf157. doi: 10.1093/jbmrpl/ziaf157 (PMC12579926; doi:10.1093/jbmrpl/ziaf157)
Supplement: FGF19_Aging_Osteoarcopenia-Zhang_Garg_ziaf157 [file fgf19_aging_osteoarcopenia-zhang_garg_ziaf157.docx]

**Supplementary Figure 1: FGF19 treatment shows no effect on atrophy and inflammation markers in muscle.** Panel of figures showing expression levels in the soleus muscle of four genes involved in muscle atrophy (A) and two genes involved in muscle inflammation (B). Expression of each gene was normalized to the housekeeping gene *TATA-box binding protein (Tbp)*. Results are expressed in arbitrary units (a.u.). Data are presented as mean ± SEM. All data were analysed using Mann-Whitney U tests. Mice treated with the vehicle solution are represented in black, while mice treated with FGF19 (0.1 mg/kg) are represented in grey. Genes analysed include *Atrogin1* (Fbxo32, F-box protein 32), *MuRF1* (Trim63, tripartite motif-containing 63), *Mstn* (Myostatin), *Fndc5* (Fibronectin type III domain containing 5), *Il6* (Interleukin 6), and *Il1b* (Interleukin 1 beta).

**
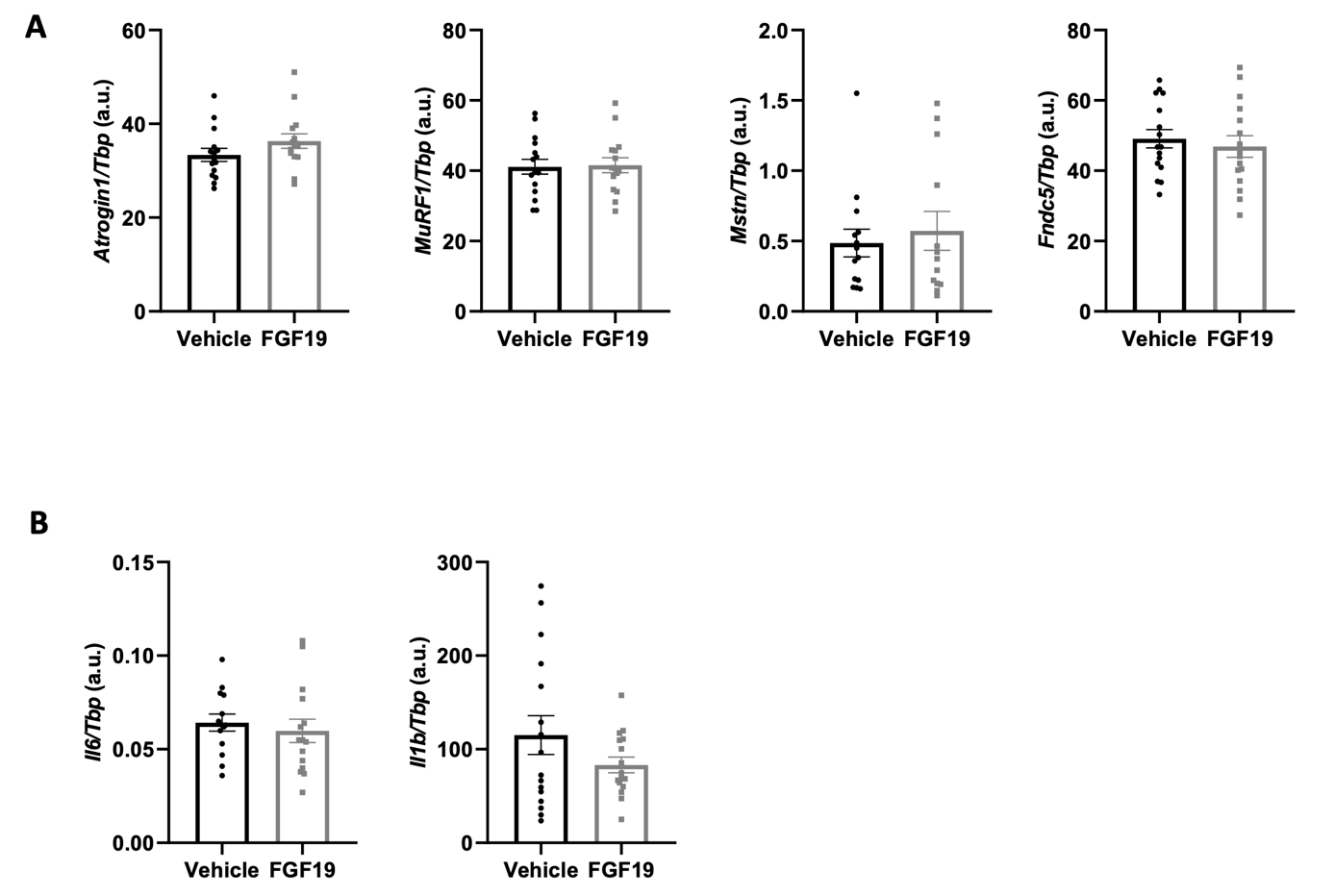
**

**Supplementary Figure 2: No effect of FGF19 treatment on bone microarchitectural cortical and trabecular parameters of the tibia. Panel of figures representing bone microarchitecture analysis of** cortical bone at **the tibia** diaphysis midshaft (A) and of trabecular bone at the tibia proximal metaphysis (B)**.** No significant difference could be recorded between vehicle-treated and FGF-treated groups: Tt.Ar (Total cross-sectional Area inside the periosteal surface); Ct.Ar (Cortical Area); Ma.Ar (Marrow Area); Ct.Po (Cortical Porosity); Ct.Th (Cortical Thickness); TMD (Tissue Mineral Density); BV/TV: Bone Volume/Total (bone volume fraction); Tb.Th (Trabecular Thickness); Tb.N (Trabecular Number); Tb.Sp (Trabecular Separation). Data are presented as mean ± SEM. All data were analysed using Mann-Whitney U tests. Mice treated with the vehicle solution are represented in black, while mice treated with FGF19 (0.1 mg/kg) are represented in grey.


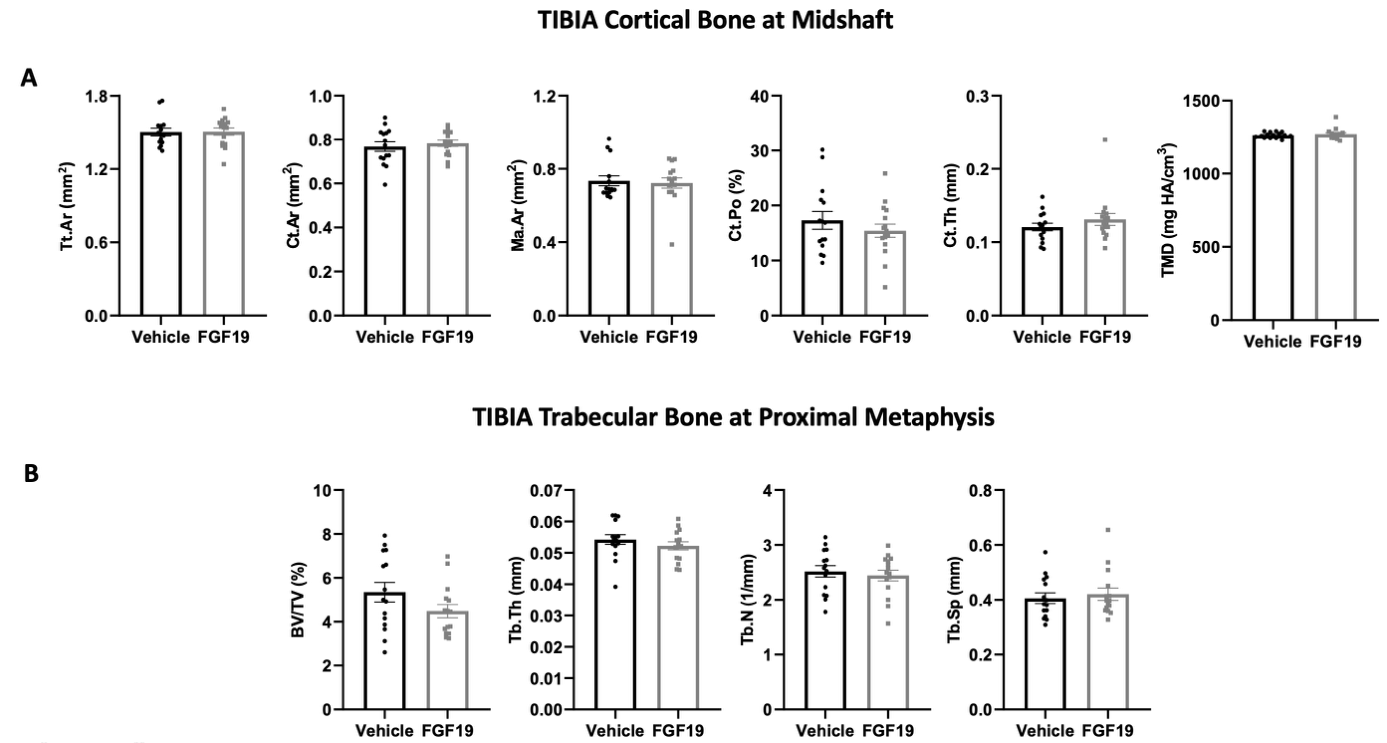
**Supplementary Figure 3: Limited effect of FGF19 treatment on bone microarchitectural parameters of the lumber vertebra L2. Panel of figures representing bone microarchitecture analysis of** trabaecular (A) and of cortical (B) bone at the second lumbar vertebra, L2**.** No significant difference could be recorded between vehicle-treated and FGF-treated groups for all tested parameters, except cortical porosity, which was lower in the treated group. BV/TV: Bone Volume/Total (bone volume fraction); Tb.Th (Trabecular Thickness); Tb.N (Trabecular Number); Tb.Sp (Trabecular Separation); Tt.Ar (Total cross-sectional Area inside the periosteal surface); Ct.Ar (Cortical Area); Ma.Ar (Marrow Area); Ct.Th (Cortical Thickness); Ct.Po (Cortical Porosity); TMD (Tissue Mineral Density). Data are presented as mean ± SEM. All data were analysed using Mann-Whitney U tests. * indicates p < 0.05. Mice treated with the vehicle solution are represented in black, while mice treated with FGF19 (0.1 mg/kg) are represented in grey.


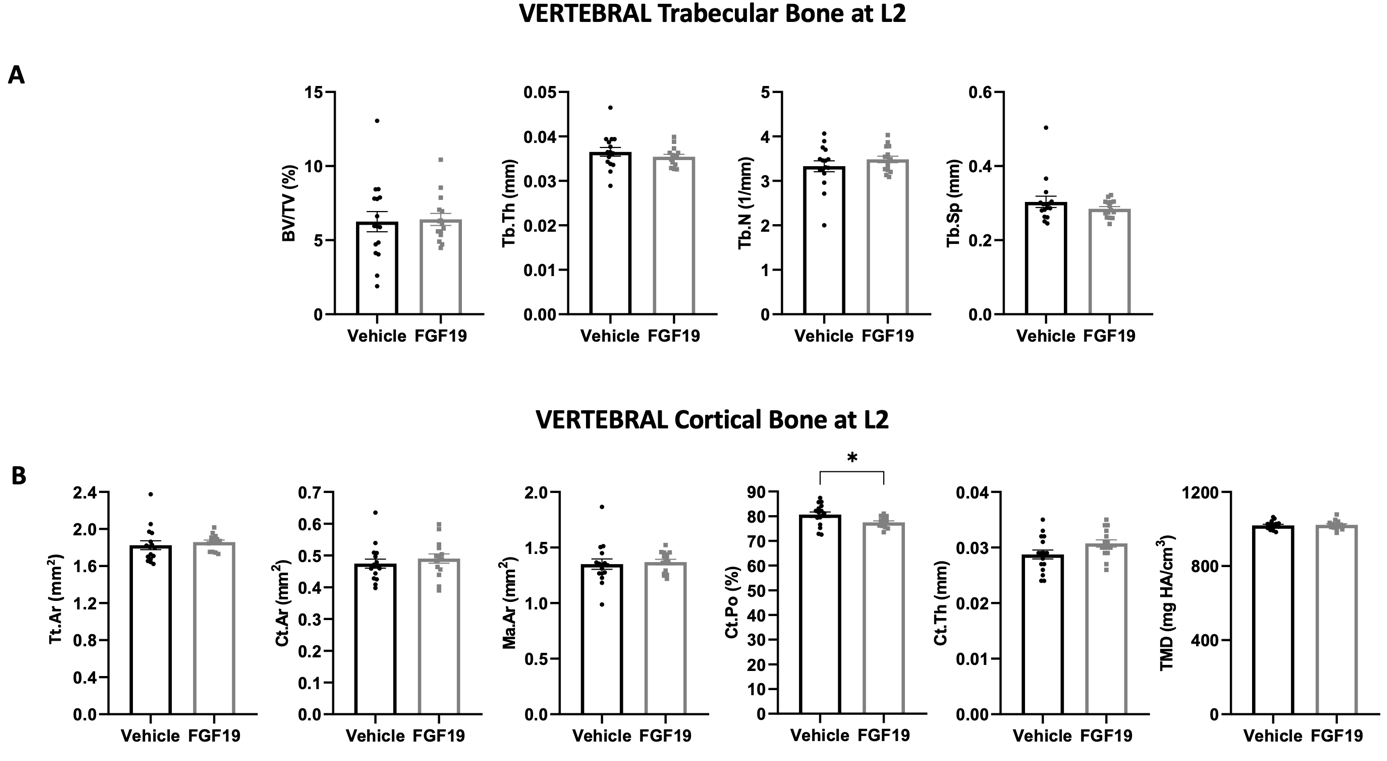
**Supplementary Figure 4: FGF19 treatment shows a very limited effect on a panel of marker genes in femur after three weeks of treatment.** Panel of figures showing expression levels in the femur of eighteen genes involved in mesenchymal stem cell and bone cell differentiation, maturation and activity: (A) bone formation initiation, mineralisation and homeostasis; (B) osteoclastogenesis, osteoclast maturation and resorption activity; (C) mesenchymal stem cell differentiation towards the adipogenic fate. Expression of each gene was normalized to the housekeeping gene *beta-actin*. And it is shown in the graphs as fold-change relative to the average of the control (vehicle) group. Genes sinclude *Runx2, Osterix/Sp7, ALP (Alkaline Phosphatase), Ocn (Osteocalcin), Opg (Osteoprotegerin), SOST (Sclerostin), c-Fos, Rank, Rank-L (RANK-Ligand), Rank-L/Opg ratio, Dscstamp, Atp6b0d2, TRAP (Tartrate-Resistant Alkaline Phosphatase), Chathepsin K, MMP9 (Matrix Metallo-Proteinase 9), PPARgamma, CEBP-alpha, CEBP-beta.* Data are presented as mean ± SEM. All data were analysed using Mann-Whitney U tests. *** indicates p < 0.001. Mice treated with the vehicle solution are represented in black, while mice treated with FGF19 (0.1 mg/kg) are represented in grey.


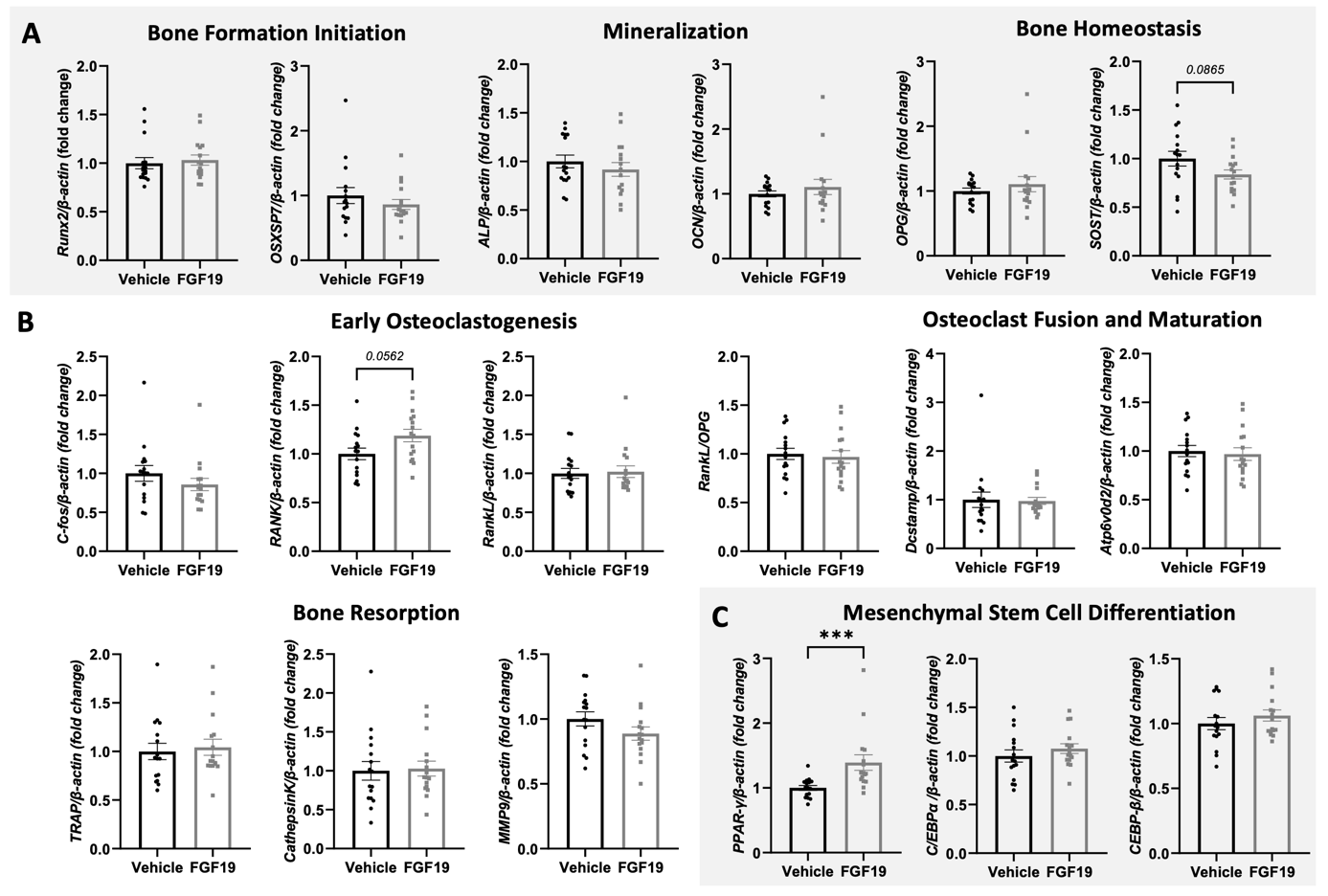


**Supplementary Table 1: Primer sequences used for quantitative PCR analysis.**

Abbreviations: *Tbp*, TATA-box binding protein; *Atrogin1 (Fbxo32)*, F-box protein 32; *MuRF1 (Trim63)*, Muscle RING-finger protein-1; *Mstn*, Myostatin; *Fndc5*, Fibronectin type III domain-containing protein 5; *Il6*, Interleukin-6; *Il1b*, Interleukin-1 beta; *β-actin*, Beta-actin; *OCN*, Osteocalcin; *OPG*, Osteoprotegerin; *OSX*, Osterix; *SOST*, Sclerostin; *Runx2*, Runt-related transcription factor 2; *ALP*, Alkaline phosphatase; *Cfos*, c-Fos proto-oncogene; *Atp6v0d2*, V-type proton ATPase subunit d2; *TRAcP*, Tartrate-resistant acid phosphatase; *RankL*, Receptor activator of nuclear factor kappa-B ligand; *Dcstamp*, Dendrocyte-expressed seven transmembrane protein; *MMP9*, Matrix metallopeptidase 9; *CTSK*, Cathepsin K; *Rank*, Receptor activator of nuclear factor kappa-B; *PPAR-γ*, Peroxisome proliferator-activated receptor gamma; *C/EBPα*, CCAAT/enhancer-binding protein alpha; *C/EBPβ*, CCAAT/enhancer-binding protein beta.

| Protein | Forward | Reverse | Gene bank ID |
| --- | --- | --- | --- |
| Tbp | TGGTGTGCACAGGAGCCAAG | TTCACATCACAGCTCCCCAC | [NM_013684.3](https://www.ncbi.nlm.nih.gov/nuccore/NM_013684.3) |
| Atrogin1 (Fbxo32) | CTCTGCCAGTACCACTTCTC | ATGGTCAGTGCCCTTCCAGG | [NM_026346.3](https://www.ncbi.nlm.nih.gov/nuccore/NM_026346.3) |
| MuRF1 (Trim63) | TGCATCTCCATGCTGGTGGC | CTTCTTCTCGTCCAGGATGG | [NM_001039048.2](https://www.ncbi.nlm.nih.gov/nuccore/NM_001039048.2) |
| Mstn | T GCTGTAACCTTCCCAGGACC | GTGCTCATCGCAGTCAAGCCC | [NM_010834.3](https://www.ncbi.nlm.nih.gov/nuccore/NM_010834.3) |
| Fndc5 | CTCTCAGCAGAAGAAGGATG | ACCACAACAATGATCAGCAC | [NM_027402.4](https://www.ncbi.nlm.nih.gov/nuccore/NM_027402.4) |
| Il6 | AGTTGCCTTCTTGGGACTGAT | TCCACGATTTCCCAGAGAAC | [NM_001314054.1](https://www.ncbi.nlm.nih.gov/nuccore/NM_001314054.1) |
| Il1b | ACTGTTCCTGAACTCAACTG | CTTGTTGATGTGCTGCTGCG | NM_008361.4 |
| β-actin | CCTCTATGCCAACACAGTGC | TCTGCTGGAAGGTGGACAGT | NM_007393.3 |
| OCN | CTCTGACCTCACAGATGCCAA | CTGGTCTGATAGCTCGTCACA | NM_007541.3 |
| OPG | TGCTGCGCACTCCTGGTGCT | CACTCCTGCTTCACGGACTG | NM_008764.3 |
| OSX | ATGGCGTCCTCTCTGCTTG | AAGGTCAGCGTATGGCTTCT | NM_130458.3 |
| SOST | TCCTCCTGAGAACAACCAGAC | TGTGAGGAAGCGGGTGTAGTG | NM_024449.5 |
| Runx2 | GACACTGCCACCTCTGACTT | GCAGGTACGTGTGGTAGTGA | NM_001145920.2 |
| ALP | AGTTACTGGCGACAGCAAGC | GGACCTGAGCGTTGGTGTTA | NM_007431.2 |
| Cfos | GCCGACTCCTTCTCCAGCAT | GGCACTAGAGACGGACAGAT | NM_010234.3 |
| Atp6v0d2 | GACACGGAGATGAGGAAGAA | GGTGACACTTGGCTAGAACT | NM_175406.3 |
| TRAcP | GGCAACGTCTCTGCACAGAT | TTGAGCCAGGACAGCTGAGT | NM_001102405.1 |
| RankL | AGCATCGCTCTGTTCCTGTA | CCATGAGCCTTCCATCATAG | NM_011613.3 |
| Dcstamp | TGTGCTTGTGGAGGAACCTA | GGATGAAGTCCAGCCAGCTA | NM_029422.4 |
| MMP9 | CCGAAGCGGACATTGTCATC | TCTGTGGTGCAGGCCGAATA | NM_013599 |
| CTSK | TGACCACTGCCTTCCAATAC | TGCCGTGGCGTTATACATAC | NM_007802.4 |
| Rank | CCAGGACAGGGCTGATGAGA | TGGCTGACATACACCACGATGA | NM_009399.3 |
| PPAR-γ | TCCGTGATGGAAGACCACTC | CCACAGACTCGGCACTCAAT | NM_001127330.1 |
| C/EBP α | CAAGCCAGGACTAGGAGATT | CCAAGGCACAAGGTTACTTC | NM_007678.3 |
| C/EBP β | GACAAGCTGAGCGACGAGTA | AGCTGCTCCACCTTCTTCTG | NM_009883.3 |
